# Association between Obesity and Circulating Brain-Derived Neurotrophic Factor (BDNF) Levels: Systematic Review of Literature and Meta-Analysis

**DOI:** 10.3390/ijms19082281

**Published:** 2018-08-03

**Authors:** Leonardo Sandrini, Alessandro Di Minno, Patrizia Amadio, Alessandro Ieraci, Elena Tremoli, Silvia S. Barbieri

**Affiliations:** 1 Dipartimento di Scienze Farmacologiche e Biomolecolari, Università degli Studi di Milano, 20133 Milan, Italy; leonardo.sandrini@unimi.it (L.S.); alessandro.ieraci@unimi.it (A.I.); 2 Centro Cardiologico Monzino IRCCS, 20138 Milan, Italy; alessandro.diminno@ccfm.it (A.D.M.); patrizia.amadio@ccfm.it (P.A.); elena.tremoli@ccfm.it (E.T.)

**Keywords:** BDNF, obesity, meta-analysis

## Abstract

Reduction in brain-derived neurotrophic factor (BDNF) expression in the brain as well as mutations in BDNF gene and/or of its receptor are associated to obesity in both human and animal models. However, the association between circulating levels of BDNF and obesity is still not defined. To answer this question, we performed a meta-analysis carrying out a systematic search in electronic databases. Ten studies (307 obese patients and 236 controls) were included in the analysis. Our data show that obese patients have levels of BDNF similar to those of controls (SMD: 0.01, 95% CI: −0.28, 0.30, *p* = 0.94). The lack of difference was further confirmed both in studies in which BDNF levels were assessed in serum (MD: −0.93 ng/mL, 95% CI: −3.34, 1.48, *p* = 0.45) and in plasma (MD: 0.15 ng/mL, 95% CI: −0.09, 0.39, *p* = 0.23). Data evaluation has shown that some bias might affect BDNF measurements (e.g., subject recruitment, procedures of sampling, handling, and storage), leading to a difficult interpretation of the results. Standardization of the procedures is still needed to reach strong, affordable, and reliable conclusions.

## 1. Introduction

Among the independent risk factors in cardiovascular disease (CVD), obesity is one of the most relevant and increasing global epidemic, leading to higher morbidity and mortality and a shortening in life expectancy [1].

Obesity is a metabolic dysfunction recently associated also with a low-grade inflammatory state [2,3]. However, these findings do not explain the full clinical picture of obesity, and new mechanisms responsible for the development and progression of this pathological condition are under investigation. In particular, factors involved in the balance of energy expenditure control lipid and glucose levels and cardiovascular homeostasis, are now considered as a new family named metabotropic factors [4]. Interestingly, nerve growth factor (NGF) and brain-derived neurotrophic factor (BDNF) have been included in this family [5].

This neurotrophin is synthetized not only in neurons but also in immune cells, adipocytes, endothelial cells, and monocytes, and its levels are detectable in different tissues including brain and blood [6,7,8].

The multifaceted role of BDNF, from his neurothrophic activity to his involvement in inflammation, metabolism [5] and cardiovascular diseases [9,10], led to coining the term ‘triactome’, that effectively explains the tight interactions between brain, immune system, and adipose tissue and their role in promoting the development of cardiometabolic diseases [11].

Actually, it has been established that hypothalamic reduction of BDNF modulates energy homeostasis affecting food intake and promoting an anorectic signal [12]. Indeed, BDNF haploinsufficiency [13,14] or missense mutations in its receptor, TrkB [15,16], are associated with hyperfagia, weight gain, and obesity both in human and in mouse models. In line with these observations, both exogenous BDNF administration and BDNF gene transfer in a mouse model of obesity and type 2 diabetes mellitus restore normal food intake, inducing weight loss and decreasing insulin resistance [17,18], which supports the concept that BDNF deficit in the brain induces a metabotropic impairment leading to obesity [19,20].

Interestingly, the positive correlation between brain and circulating BDNF suggests that BDNF levels in blood reflect the levels occurring in the central nervous system (CNS) [21]. Thus, circulating BDNF has been proposed as a potential biomarker for neuro-psychiatric disorders and neurodegenerative diseases [22,23,24,25,26,27]. On the contrary, the relationship between circulating BDNF and cardiometabolic disorders is still unclear.

In this context, a significant reduction of circulating BDNF levels in obese subjects versus controls was found [28,29], suggesting a negative association between circulating BDNF and this pathology [11,19]. However, in the last few years this relationship was called into question. Indeed, different studies have shown either that circulating BDNF levels are higher in obese subjects than in controls [9,30] or that there are no differences between the two groups [31]. In order to disentangle these controversies regarding the association between circulating levels of BDNF and obesity, we performed a systematic review and meta-analysis of the literature.

## 2. Results

### 2.1. Literature Search Process

**Figure 1:**
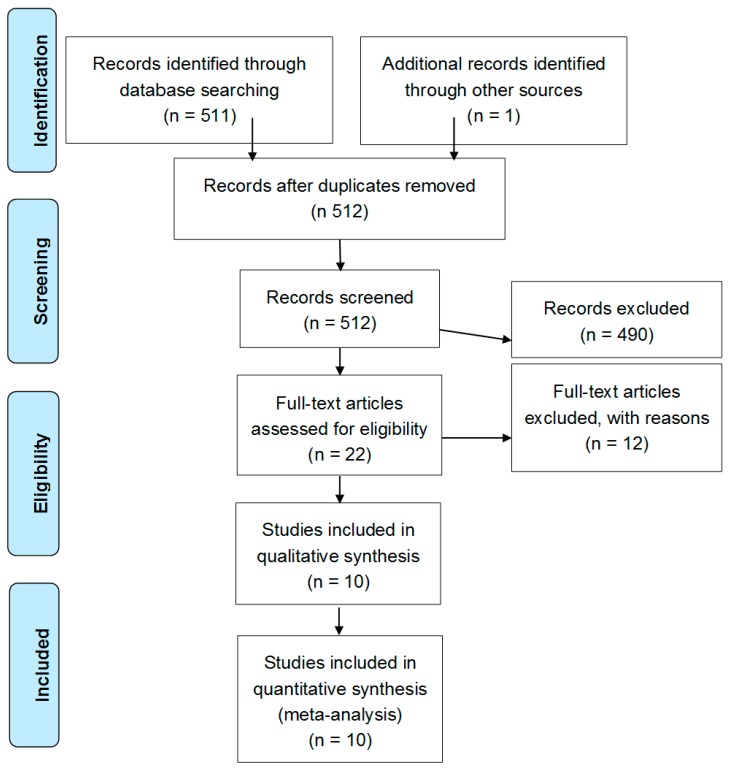
Preferred reporting items for systematic reviews and meta-analyses (PRISMA) flow diagram of literature search process and result.

After excluding duplicate results, the search retrieved 512 articles. Of these studies, 490 were excluded because they were off the topic after scanning the title and/or the abstract and because they were reviews/comments/case reports or they lacked data of interest. A total of 12 studies were excluded after full-length paper evaluation. Thus, 10 studies on 307 obese patients and 236 controls were included in the final analysis (Figure 1). In detail, we include three studies with children (94 cases and 71 controls), and seven with the elderly (213 cases and 165 controls).

### 2.2. Study Characteristics

Table 1 and Table S1 describes the major characteristics of included studies. A total of five studies were case-control, four had a prospective design, and one was cross-sectional.

The number of patients varied from 7 to 73, the mean age from 7.9 to >65 years, and the prevalence of male gender from 0% to 100%. Mean body mass index (BMI) varied from 22.5 Kg/m^2^ to 34.00 Kg/m^2^, mean waist circumference from 72.00 cm to 98.10 cm. Mean total cholesterol level varied from 156.40 mg/dL to 233.33 mg/dL, HDL cholesterol varied from 36.91 mg/dL to 56.86 mg/dL, LDL cholesterol varied from 89.23 mg/dL to 155.27 mg/dL and triglycerides varied from 98.17 mg/dL to 207.00 mg/dL. Mean glycaemia varied from 83.54 mg/dL to 102.53 mg/dL.

**Figure 2:**
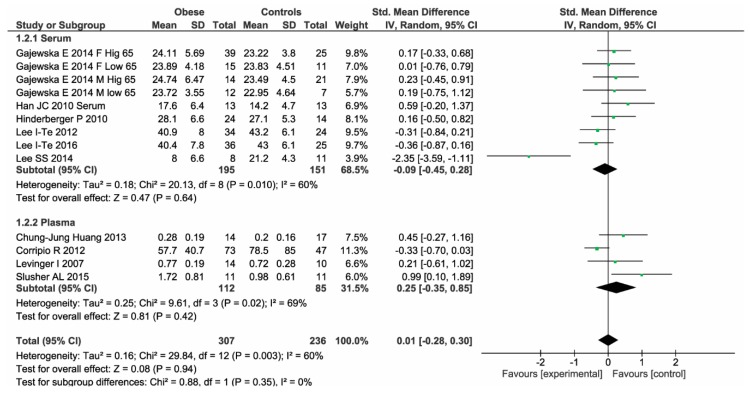
Brain derived neurotrophic factor (BDNF) levels in obese patients and controls.

In the 10 studies, we found that 307 obese patients have similar BDNF levels compared to 236 controls (SMD: 0.01, 95% CI: −0.28, 0.30, *p*-over = 0.94, I^2^ = 60%, *p*-heter = 0.003) (Figure 2). As shown in Table 2, the lack of difference was consistently confirmed both in studies in which BDNF levels were assessed in serum (MD: −0.93 ng/mL, 95% CI: −3.34, 1.48, *p*-over = 0.45, I^2^ = 74%, *p*-heter = 0.0001) and in studies using plasma samples (MD: 0.15 ng/mL, 95% CI: −0.09, 0.39, *p*-over = 0.23, I^2^ = 58%, *p*-heter = 0.07).

**Figure 3:**
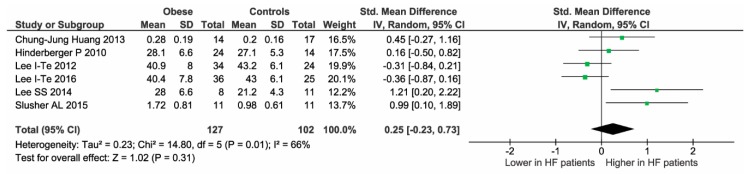
Sensitivity analysis after excluding the four low-quality studies.

No heterogeneity reduction was observed even after excluding one study at time. The median value of NOS quality assessment was 3. Thus, four studies were considered “low quality” (NOS < 3) (Table 3). Of interest, after excluding these studies, all results were entirely confirmed (Figure 3).

### 2.3. Meta-Regression Analyses

**Figure 4:**
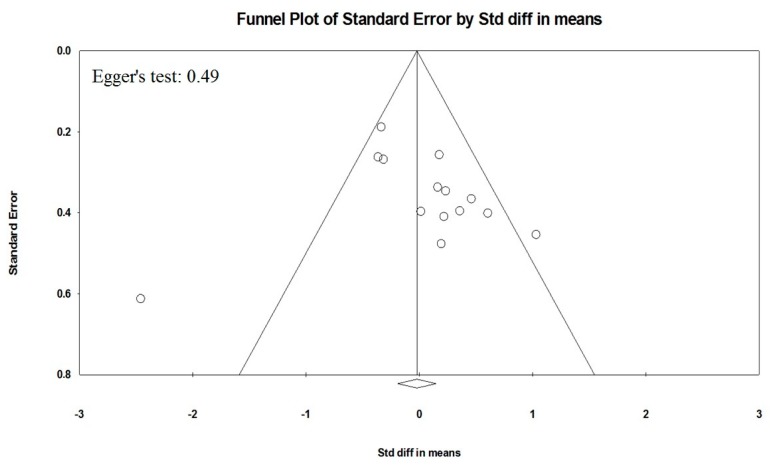
Publication bias. Funnel plots of effect size versus standard error for studies evaluating BDNF levels in obese patients and controls.

Meta-regression models showed that cardiovascular (CV) risk factors (hypertension, diabetes, hyperlipidemia, and smoking habit) (Figure S1) and demographic variables (male gender and age) (Figure S2) did not influence the association between BDNF levels and obesity. Because it is recognized that publication bias affects the results of meta-analyses, we attempted to assess this potential bias using funnel plot analysis. Funnel plots of effect size versus standard error for studies evaluating levels of BDNF in obese patients and controls were rather asymmetrical, and the Egger test confirmed the absence of a significant publication bias (Egger’s *p* = 0.49, Figure 4).

## 3. Discussion

To the best of our knowledge, this is the first meta-analysis investigating the association between circulating levels of BDNF and obesity. The current meta-analysis is not able to find any association between BDNF levels, both in plasma and in serum, and obesity. These findings were further confirmed by the sensitivity analysis, suggesting that at the current state of the art there is no evidence regarding this association.

The current opinion that obesity is associated with lower levels of circulating BDNF [11,19], and that restoring its physiological levels by administration of exogenous BDNF may prevent the detrimental effect of the metabolic syndrome [11] should be considered carefully.

Indeed, whereas the correlation between obesity and hypothalamic BDNF reduction in both human and animal models [40,41,42,43] is well established, the association of this pathology with circulating BDNF derives predominantly only from the assumption that circulating BDNF mirrors the one in the brain. It has been hypothesized that BDNF from the brain moves into circulation after crossing the blood–brain barrier [21]. However, the hypothesis that circulating BNDF derives only from brain is now under debate, and although, vascular endothelium [8] has been proposed as a source of circulating BDNF, its origins are still poorly understood [44,45].

BDNF is detectable in plasma even if its levels are 100 to 200-fold lower in plasma than those of serum. Plasma BDNF levels change within a day and can be influenced by environmental factors and correlate positively with platelets activation state [46,47,48] and might well reflect the physio-pathological condition of the body [49]. However, plasma BDNF is quite unstable [50] and sensitive to preparation procedure [51,52,53].

On the other hand, serum BDNF mostly reflects the total amount of this neurotrophin released from the alpha granules of platelets after blood clotting [47]. Serum BDNF is more stable, also after long term storage [51] but its amount in serum is modified by temperature and time of clotting [46].

According to other studies [30,31], we did not find a significant difference among the analyzed groups.

However, the results here obtained might be influenced by potential limitations that are herein critically discussed.

First, criteria for patient or control recruitment frequently do not take into account parameters and/or factors that affect BDNF measurements [54,55,56]. Among these, we can find metabolic dysfunctions as well as smoke history, sex, age, and ethnicity. It is important to consider that history of previous smoking has a recognized impact on circulating BDNF levels [48]. Therefore, smokers should be excluded from studies assessing circulating levels of the protein. Interestingly, only in one study [30] was it clearly declared that smoke history represented one of the exclusion criteria used.

In addition, since it has been reported that plasma BDNF levels differ between females and males both in plasma and serum [44,49], the high variability in the percentage of male in the studies (ranging from 0 to 100%) have an impact on the results.

Three studies were carried out in children and/or adolescents. Since it has been reported that hormonal status influences circulating BDNF [57], evaluation of BDNF concentrations might be interpreted with age-specific standard. In line with this, we cannot exclude an impact of data deriving from prepubertal patients and controls on our results. 

In this meta-analysis we included in the analysis studies with both people of Western European descent and Asian subjects, even if it is known that these two ethnic groups have different circulating levels of BDNF and pro-BDNF [58]. Similarly, differences in allelic prevalence of BDNF polymorphisms that might affect protein circulating levels and are associated with obesity [59] were found among ethnic groups [60].

Based on these considerations, we performed a meta-regression analyses to assess if differences in the effect size among included studies may be affected by demographic variables (e.g., age and gender) (Figure S2), coexistence of traditional CV risk factors (e.g., hypertension, smoking habit, hyperlipidemia, and diabetes) (Figure S1). However, these factors and variables did not influence the association between BDNF levels and obesity previously observed.

In addition, procedures of sampling, handling, and storage are not always reported (Table 4), making difficult to critically analyze the data provided.

In particular, the high variability of plasma levels (ranging from 0.2017 ± 0.04198 to 57.7 ± 40.7 ng/mL) and serum (ranging from 8.0 ± 6.6 to 43.2 ± 6.1 ng/mL) of BDNF represents a major bias for our analysis. Of note, the value of plasma BDNF reported by Corripio et al. (57.70 ± 40.70 ng/mL for cases and 78.50 ± 85.00 ng/mL for obese) is about 50-fold higher than that reported by the other studies, and surprisingly it is similar to the levels reported in serum [44,49].

Concerning methodological aspects, it is well-known that different anticoagulants, temperatures, and delays in sample centrifugation, as well as conditions and time of sample storage influence the measurements of BDNF [46,51,53,61]. Similarly, the ELISA kits used to measure BDNF have different performance in terms of detection range, sensitivity, and inter-assay variations [62]. In the studies included in our meta-analysis, all the authors correctly specified the producers; however, it cannot be excluded that inter-kit variability plays an important role in BDNF measurements.

Finally, information about the effects of fasting or fed conditions were often not available even if it has been shown that diet affects circulating BDNF [63].

## 4. Materials and Methods

In order to provide a comprehensive search and analysis of data we followed a standardized research protocol already performed by our group [64]. Briefly, prospectively developed, detailing the specific objectives, the criteria for study selection, the approach to assess study quality, outcomes, and statistical methods.

### 4.1. Search Strategy

To identify all available studies, a detailed search pertaining to BDNF levels and obesity was conducted according to PRISMA (preferred reporting items for systematic reviews and meta-analyses) guidelines [65]. A systematic search was performed in the electronic databases (PubMed, Web of Science, Scopus, EMBASE), using the following search terms in all possible combinations: brain derived neurotrophic factor, BDNF, obesity, overweight, body mass index (BMI). The last search was performed on 11 June 2018. The search strategy was developed without any language or publication year restriction.

In addition, the reference lists of all retrieved articles were manually reviewed. In case of missing data, study authors were contacted by e-mail to try to retrieve original data. Two independent authors (L.S. and P.A.) analyzed each article and performed the data extraction independently. In case of disagreement, a third investigator was consulted (A.D.M). Discrepancies were resolved by consensus. Selection results showed a high inter-reader agreement (κ = 0.96) and have been reported according to PRISMA flowchart (Figure 1).

### 4.2. Data Extraction and Quality Assessment

According to the pre-specified protocol, all studies evaluating BDNF levels in obesity patients were included. Case-reports, reviews and animal studies were excluded.

To be included in the analysis, a study had to provide levels of BDNF in obese patients (BMI > 30) and controls. We also included the studies of Lee SS et al. and Corripio et al [35,37] that involved obese children with BMI < 30 since the authors stated that the patients were obese according to the guidelines.

To allow for a pooled analysis of data, studies dosing BDNF levels on samples different from plasma or serum (i.e., histological samples), lacking of a control group, including a study population with a clinical condition other than obesity were excluded. 

In each study, data regarding sample size, major clinical and demographic variables of patients, and controls and type of BDNF measurement (different commercially available kits for the dosage of BDNF) were extracted.

### 4.3. Statistical Analysis and Risk of Bias Assessment

Statistical analysis was carried out using the Review Manager software (Version 5.2, The Cochrane Collaboration, Copenhagen, Denmark). Because of the heterogeneity in the methods used for measuring BDNF in included studies, differences among cases and controls were expressed as standard mean difference (SMD) with pertinent 95% confidence intervals (95% CI). According to widely accepted guidelines, SMD is considered small if ranging from 0.2 to 0.5, medium if 0.5–0.8 and large if >0.8 [66]. When separately assessing, studies in which BDNF levels were evaluated in plasma and serum, differences among cases and controls were expressed as mean difference (MD) with 95% CI. The overall effect was tested using Z scores and significance was set at *p* < 0.05. Statistical heterogeneity between studies was assessed with chi square Cochran’s Q test and with I^2^ statistic, which measures the inconsistency across study results and describes the proportion of total variation in study estimates, that is due to heterogeneity rather than sampling error. In detail, I^2^ values of 0% indicates no heterogeneity, 25% low, 25–50% moderate, and 50% high heterogeneity [67]. Publication bias was assessed by the Egger’s test and represented graphically by funnel plots of the standard difference in means versus the standard error (Figure 4). Visual inspection of funnel plot asymmetry was performed to address for possible small-study effect, and Egger’s test was used to assess publication bias, over and above any subjective evaluation. A *p* < 0.10 was considered statistically significant [68]. In case of a significant publication bias, the Duval and Tweedie’s trim and fill method was used to allow for the estimation of an adjusted effect size [69]. In order to be as conservative as possible, the random-effect method was used for all analyses to take into account the variability among included studies.

### 4.4. Meta Regression Analyses

We hypothesized that differences in the effect size among included studies may be affected by demographic variables (mean age, male gender) and coexistence of traditional CV risk factors (hypertension, smoking habit, hyperlipidemia, diabetes). To assess the possible effect of such variables in explaining different results observed across studies, we planned to perform meta-regression analyses after implementing a regression model with BDNF levels as a dependent variable(*y*)—expressed as standardized mean difference—and the above-mentioned co-variates as independent variables (*x*). This analysis was performed with comprehensive meta-analysis (Version 2, Biostat, Englewood, NJ, USA, (2005)).

### 4.5. Sensitivity Analysis

In the frame of a sensitivity analysis, we repeated analyses by including only the studies judged as “high quality” according to Newcastle–Ottawa scale (NOS) [70]. Briefly, we judged each study by its quality by awarding a star for each item that was present. Studies reaching a value of NOS ≥ to the median value found among all the studies were included for sensitivity analysis.

## 5. Conclusions

In conclusion, our meta-analysis shows that obesity is apparently not associated with lower levels of circulating BDNF. However, considerations regarding the limitations deriving from criteria of subject selection and methodological aspects are often underestimated. This leads to a difficult interpretation of the results and makes it more difficult to reach strong, affordable, and reliable conclusions when comparing data from studies present in the literature.

## Figures and Tables

**Table 1:** Demographic and clinical data of obese patients and healthy controls of the included studies.

Author, Country	Study Design	Type of Pathology	BDNF Measurement	Cases (n)	Controls (n)	BDNF (Ng/ML) Cases Mean	BDNF (ng/mL) Controls Mean	BDNF (ng/mL) Weighted Average	Age (Years) Cases	Age (Years) Controls	Age (Years) Mean	Males (%)	BMI (kg/m^2^) Cases	BMI (kg/m^2^) Controls	BMI (kg/m^2^) Mean	WC (cm) Cases	WC (cm) Controls	WC (cm) Mean
Lee I-Te 2016, Taiwan [32]	Prospective	Obesity and MetS	Serum (ELISA)	36	25	40.40	43.00	41.47	44	39	41.95	100.0	33.5	22.5	29.0	109.1	81.9	98
Slusher AL 2015, United States [30]	Case-control/Prospective	Obesity	Plasma (ELISA)	11	11	1.72	0.98	1.35	22.91	23.27	23.09	40.9	35.72	21.89	28.8	99.32	71.36	85.3
Hinderberger P 2016, Germany [31]	Case-control	Obesity	Serum (ELISA)	24	14	28.10	27.10	27.73	36.9	36.1	36.61	26.3	40.7	22.5	34.0	ND	ND	ND
Gajewska E 2014, Poland [33]	Case-control	Obesity	Serum (ELISA)	15	11	23.89	23.83	23.86	≤65	≤65	≤65	0	>40	ND	ND	ND	ND	ND
				39	25	24.11	23.22	23.76	>66	>66	>66	0	>40	ND	ND	ND	ND	ND
				12	7	23.72	22.95	23.44	≤65	≤65	≤65	100	>40	ND	ND	ND	ND	ND
				14	21	24.74	23.94	23.99	>66	>66	>66	100	>40	ND	ND	ND	ND	ND
Han JC 2010, United States [34]	Cross-sectional	Obesity	Serum (ELISA)	13	13	17.60	14.20	15.90	12.3	12.4	12.35	92	33.1	17.2	25.2	ND	ND	ND
Corripio R 2012, Spain [35]	Prospective	Obesity	Plasma (ELISA)	73	47	57.70	78.50	65.85	8.03	7.74	7.92	55.8	26.5	16.2	22.5	81.3	57.5	72.0
Lee I-Te 2012, Taiwan [36]	Case-control	Obesity and MetS	Serum (ELISA)	34	24	40.90	43.20	41.85	41	39	40.17	100	33.9	22.5	29.2	109.3	82.2	98.1
Lee SS 2014, Korea [37]	Prospective	Obesity	Serum (ELISA)	8	11	8.00	21.20	15.64	16.3	16.4	16.36	65	27.47	22.35	24.5	ND	ND	ND
Levinger I 2007, Australia [38]	Prospective	Obesity and MetS	Plasma (ELISA)	14	10	1.03	0.70	0.89	51.9	48.9	50.65	54	30.3	23.8	27.6	99.4	81	91.7
Chun-Jung Huang 2014, United States [39]	Case-control	Obesity	Plasma (ELISA)	14	17	0.28	0.20	0.24	22.64	22.94	22.80	35	38.18	21.21	28.9	111.46	68.74	88.0

WC: waist circumference; BMI: body mass index; ND: not declared.

**Table 2:** Subgroup analysis: stratification of the analysis according to different BDNF matrices used for BDNF measurements.

	Number of Studies	Number of Patients	Effect Size	Test for Subgroup Differences
Serum	6	195 cases 151 controls	MD: −0.93; 95% CI: −3.34, 1.48, *p* = 0.45, I^2^: 74%, *p* = 0.0001	Chi^2^: 0.88, *p* = 0.35
Plasma	4	112 cases 85 controls	MD: 0.15; 95% CI: −0.09, 0.39, *p* = 0.23, I^2^: 58%, *p* = 0.07	

MD: Mean Difference; CI: Confidence Interval; Chi^2^: chi-squared.

**Table 3:** Quality assessment (Newcastle–Ottawa scale) of 10 studies.

	Definition of Cases	Representativeness of Cases	Selection of Controls	Definition of Controls	Comparability of Cases and Controls	Same Method of Ascertainment	Quality
Lee I-Te, 2016 [31]	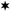	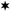	-	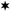	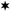	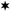	5
Slusher AL, 2015 [29]	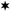	-	-	-	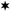	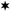	3
Hinderberger P, 2016 [32]	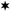	-	-	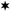	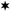	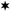	4
Gajewska E, 2014 [33]	-	-	-	-	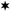	-	1
Han JC, 2010 [34]	-	-	-	-	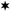	-	1
Corripio R, 2012 [35]	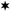	-	-	-	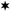	-	2
Lee I-TE, 2012 [36]	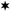	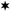	-	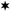	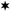	-	4
Lee SS, 2014 [37]	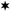	-	-	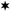	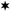	-	3
Levinger I, 2007 [38]	-	-	-	-	-	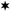	1
Chun-Jung Huang 2013 [39]	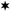	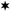	-	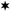	-	-	3

**Table 4:** Characteristics regarding procedures of sampling, patients condition, handling, storage, and measurements of BDNF.

Author	Sample	Information Reported about Blood Collection	Patients Condition	Information Reported about Procedure to Obtain Plasma/Serum	Conservation	Assay Kit
Lee I-Te 2016 [31]	Serum	ND	ND	ND	ND	ELISA Quantikine; R&D Systems, Inc., Minneapolis, MN
Slusher AL 2015 [29]	Plasma	Blood collected in EDTA tubes (BD vacutainer)	ND	Immediately centriguged at 1000× *g* for 20 min at room temperature	−80 °C until measurements (no reported storage time)	Promega, Madison, WI
Hinderberger P 2016 [32]	Serum	Blood collected in two timeframes: 8–10 a.m. and 2–4 p.m.	ND	2 h of clotting	ND	ELISA Quantikine; R&D Systems, Inc., Minneapolis, MN
Gajewska E 2014 [33]	Serum	ND	ND	ND	ND	ELISA Quantikine; R&D Systems, Inc., Minneapolis, MN
Han JC 2010 [34]	Serum	Serum obtained using serum separator tubes	ND	30 min of clotting	−70 °C until measurements (no reported storage time)	ELISA Quantikine; R&D Systems, Inc., Minneapolis, MN
Corripio R 2012 [35]	Plasma	ND	Samples obtained after 12 h of fasting	ND	−80 °C until measurements (no reported storage time)	Human BDNF ELISA Kit, RayBiotech, Inc, Norcross, GA
Lee I-TE 2012 [36]	Serum	ND	ND	ND	ND	ELISA Quantikine; R&D Systems, Inc., Minneapolis, MN
Lee SS 2014 [37]	Serum	ND	Samples obtained after 12 h of fasting	Centrifugation at 3000 rpm for 10 min	−80 °C until measurements (no reported storage time)	ELISA Quantikine; R&D Systems, Inc., Minneapolis, MN
Levinger I 2007, Australia [38]	Plasma	ND	Samples obtained after 3 h of fasting	ND	−20 °C until measurements (no reported storage time)	ELISA Quantikine; R&D Systems, Inc., Minneapolis, MN
Chun-Jung Huang 2013 [39]	Plasma	Blood collected in EDTA tubes	ND	Centriguged at 2000× *g* for 15 min at 4 °C	−80 °C until measurements (no reported storage time)	Abcam, Cambridge, MA

ND: Not Declared.

## Supplementary Materials

Supplementary materials can be found at http://www.mdpi.com/1422-0067/19/8/2281/s1. Figure S1: Scatterplot of the relationship between hypertension, diabetes, hyperlipidemia and smoking habit and standard mean difference. Figure S2: Scatterplot of the relationship between male gender and age and mean difference. Table S1: Demographic and clinical data of obese patients and healthy controls of the included studies.

## Abbreviations

BDNF	brain-derived neurotrophic factor
BMI	body mass index
CI	confidence interval
CNS CV	central nervous system cardiovascular
CVD	cardiovascular disease
ELISA	enzyme-linked immunosorbent assay
MD	mean difference
NOS	Newcastle–Ottawa scale
SMD	standard mean difference

## References

[1] Payne R.A. (2012). Cardiovascular risk. Br. J. Clin. Pharmacol..

[2] Monteiro R., Azevedo I. (2010). Chronic inflammation in obesity and the metabolic syndrome. Mediat. Inflamm..

[3] Donath M.Y., Shoelson S.E. (2011). Type 2 diabetes as an inflammatory disease. Nat. Rev. Immunol..

[4] Fiore M., Chaldakov G.N., Rancic G., Bełtowski J., Tunçel N., Aloe L. (2014). An integrated view: Neuroadipocrinology of diabesity. Serbian J. Exp. Clin. Res..

[5] Chaldakov G. (2011). The metabotrophic ngf and BDNF: An emerging concept. Arch. Italiennes Biol..

[6] Kerschensteiner M., Gallmeier E., Behrens L., Leal V.V., Misgeld T., Klinkert W.E., Kolbeck R., Hoppe E., Oropeza-Wekerle R.L., Bartke I. (1999). Activated human T cells, B cells, and monocytes produce brain-derived neurotrophic factor in vitro and in inflammatory brain lesions: A neuroprotective role of inflammation?. J. Exp. Med..

[7] Sornelli F., Fiore M., Chaldakov G.N., Aloe L. (2009). Adipose tissue-derived nerve growth factor and brain-derived neurotrophic factor: Results from experimental stress and diabetes. Gen. Physiol. Biophys..

[8] Nakahashi T., Fujimura H., Altar C.A., Li J., Kambayashi J., Tandon N.N., Sun B. (2000). Vascular endothelial cells synthesize and secrete brain-derived neurotrophic factor. FEBS Lett..

[9] Golden E., Emiliano A., Maudsley S., Windham B.G., Carlson O.D., Egan J.M., Driscoll I., Ferrucci L., Martin B., Mattson M.P. (2010). Circulating brain-derived neurotrophic factor and indices of metabolic and cardiovascular health: Data from the baltimore longitudinal study of aging. PLoS ONE.

[10] Amadio P., Colombo G.I., Tarantino E., Gianellini S., Ieraci A., Brioschi M., Banfi C., Werba J.P., Parolari A., Lee F.S. (2017). Bdnfval66met polymorphism: A potential bridge between depression and thrombosis. Eur. Heart J..

[11] Singh R.B., Takahashi T., Tokunaga M., Wilczynska A., Kim C.J., Meester F.D., Handjieva-Darlenska T., Cheema S.K., Wilson D.W., Milovanovic B. (2014). Effect of brain derived neurotrophic factor, in relation to diet and lifestyle factors, for prevention of neuropsychiatric and vascular diseases and diabetes. Open Nutr. J..

[12] Lebrun B., Bariohay B., Moyse E., Jean A. (2006). Brain-derived neurotrophic factor (BDNF) and food intake regulation: A minireview. Auton Neurosci..

[13] Kernie S.G., Liebl D.J., Parada L.F. (2000). Bdnf regulates eating behavior and locomotor activity in mice. EMBO J..

[14] Lyons W.E., Mamounas L.A., Ricaurte G.A., Coppola V., Reid S.W., Bora S.H., Wihler C., Koliatsos V.E., Tessarollo L. (1999). Brain-derived neurotrophic factor-deficient mice develop aggressiveness and hyperphagia in conjunction with brain serotonergic abnormalities. Proc. Natl. Acad. Sci. USA.

[15] Xu B., Goulding E.H., Zang K., Cepoi D., Cone R.D., Jones K.R., Tecott L.H., Reichardt L.F. (2003). Brain-derived neurotrophic factor regulates energy balance downstream of melanocortin-4 receptor. Nat. Neurosci..

[16] Yeo G.S., Connie Hung C.C., Rochford J., Keogh J., Gray J., Sivaramakrishnan S., O’Rahilly S., Farooqi I.S. (2004). A de novo mutation affecting human trkb associated with severe obesity and developmental delay. Nat. Neurosci..

[17] Cao L., Lin E.J., Cahill M.C., Wang C., Liu X., During M.J. (2009). Molecular therapy of obesity and diabetes by a physiological autoregulatory approach. Nat. Med..

[18] Bariohay B., Lebrun B., Moyse E., Jean A. (2005). Brain-derived neurotrophic factor plays a role as an anorexigenic factor in the dorsal vagal complex. Endocrinology.

[19] Motamedi S., Karimi I., Jafari F. (2017). The interrelationship of metabolic syndrome and neurodegenerative diseases with focus on brain-derived neurotrophic factor (BDNF): Kill two birds with one stone. Metab. Brain Dis..

[20] Das U.N. (2010). Obesity: Genes, brain, gut and environment. Nutrition.

[21] Klein A.B., Williamson R., Santini M.A., Clemmensen C., Ettrup A., Rios M., Knudsen G.M., Aznar S. (2011). Blood BDNF concentrations reflect brain-tissue BDNF levels across species. Int. J. Neuropsychopharmacol..

[22] Polyakova M., Stuke K., Schuemberg K., Mueller K., Schoenknecht P., Schroeter M.L. (2015). Bdnf as a biomarker for successful treatment of mood disorders: A systematic & quantitative meta-analysis. J. Affect. Disord..

[23] Fernandes B.S., Steiner J., Berk M., Molendijk M.L., Gonzalez-Pinto A., Turck C.W., Nardin P., Gonçalves C.A. (2015). Peripheral brain-derived neurotrophic factor in schizophrenia and the role of antipsychotics: Meta-analysis and implications. Mol. Psychiatry.

[24] Sanada K., Zorrilla I., Iwata Y., Bermúdez-Ampudia C., Graff-Guerrero A., Martínez-Cengotitabengoa M., González-Pinto A. (2016). The efficacy of non-pharmacological interventions on brain-derived neurotrophic factor in schizophrenia: A systematic review and meta-analysis. Int. J. Mol. Sci..

[25] Cui H., Jin Y., Wang J., Weng X., Li C. (2012). Serum brain-derived neurotrophic factor (BDNF) levels in schizophrenia: A systematic review. Shanghai Arch. Psychiatry.

[26] Green M.J., Matheson S.L., Shepherd A., Weickert C.S., Carr V.J. (2011). Brain-derived neurotrophic factor levels in schizophrenia: A systematic review with meta-analysis. Mol. Psychiatry.

[27] Fernandes B.S., Molendijk M.L., Köhler C.A., Soares J.C., Leite C.M., Machado-Vieira R., Ribeiro T.L., Silva J.C., Sales P.M., Quevedo J. (2015). Peripheral brain-derived neurotrophic factor (BDNF) as a biomarker in bipolar disorder: A meta-analysis of 52 studies. BMC Med..

[28] Roth C.L., Elfers C., Gebhardt U., Müller H.L., Reinehr T. (2013). Brain-derived neurotrophic factor and its relation to leptin in obese children before and after weight loss. Metabolism.

[29] Celik Guzel E., Bakkal E., Guzel S., Eroglu H.E., Acar A., Kuçukyalcin V., Topcu B. (2014). Can low brain-derived neurotrophic factor levels be a marker of the presence of depression in obese women?. Neuropsychiatr. Dis. Treat..

[30] Slusher A.L., Whitehurst M., Zoeller R.F., Mock J.T., Maharaj A., Huang C.J. (2015). Brain-derived neurotrophic factor and substrate utilization following acute aerobic exercise in obese individuals. J. Neuroendocrinol..

[31] Hinderberger P., Rullmann M., Drabe M., Luthardt J., Becker G.A., Blüher M., Regenthal R., Sabri O., Hesse S. (2016). The effect of serum BDNF levels on central serotonin transporter availability in obese versus non-obese adults: A [(11)c]dasb positron emission tomography study. Neuropharmacology.

[32] Lee I.T., Wang J.S., Fu C.P., Lin S.Y., Sheu W.H. (2016). Relationship between body weight and the increment in serum brain-derived neurotrophic factor after oral glucose challenge in men with obesity and metabolic syndrome: A prospective study. Medicine.

[33] Gajewska E., Sobieska M., Łojko D., Wieczorowska-Tobis K., Suwalska A. (2014). Obesity itself does not influence BDNF serum levels in adults. Eur. Rev. Med. Pharmacol. Sci..

[34] Han J.C., Muehlbauer M.J., Cui H.N., Newgard C.B., Haqq A.M. (2010). Lower brain-derived neurotrophic factor in patients with prader-willi syndrome compared to obese and lean control subjects. J. Clin. Endocrinol. Metab..

[35] Corripio R., Gónzalez-Clemente J.M., Jacobo P.S., Silvia N., Lluis G., Joan V., Assumpta C. (2012). Plasma brain-derived neurotrophic factor in prepubertal obese children: Results from a 2-year lifestyle intervention programme. Clin. Endocrinol..

[36] Lee I.T., Lee W.J., Tsai I.C., Liang K.W., Lin S.Y., Wan C.J., Fu C.P., Sheu W.H. (2012). Brain-derived neurotrophic factor not associated with metabolic syndrome but inversely correlated with vascular cell adhesion molecule-1 in men without diabetes. Clin. Chim. Acta Int. J. Clin. Chem..

[37] Lee S.S., Yoo J.H., Kang S., Woo J.H., Shin K.O., Kim K.B., Cho S.Y., Roh H.T., Kim Y.I. (2014). The effects of 12 weeks regular aerobic exercise on brain-derived neurotrophic factor and inflammatory factors in juvenile obesity and type 2 diabetes mellitus. J. Phys. Ther. Sci..

[38] Levinger I., Goodman C., Matthews V., Hare D.L., Jerums G., Garnham A., Selig S. (2008). Bdnf, metabolic risk factors, and resistance training in middle-aged individuals. Med. Sci. Sports Exerc..

[39] Huang C.J., Mari D.C., Whitehurst M., Slusher A., Wilson A., Shibata Y. (2014). Brain-derived neurotrophic factor expression ex vivo in obesity. Physiol. Behav..

[40] Jin Y.J., Cao P.J., Bian W.H., Li M.E., Zhou R., Zhang L.Y., Yang M.Z. (2015). Bdnf levels in adipose tissue and hypothalamus were reduced in mice with msg-induced obesity. Nutr. Neurosci..

[41] Fox E.A., Biddinger J.E., Jones K.R., McAdams J., Worman A. (2013). Mechanism of hyperphagia contributing to obesity in brain-derived neurotrophic factor knockout mice. Neuroscience.

[42] Han J.C., Liu Q.R., Jones M., Levinn R.L., Menzie C.M., Jefferson-George K.S., Adler-Wailes D.C., Sanford E.L., Lacbawan F.L., Uhl G.R. (2008). Brain-derived neurotrophic factor and obesity in the wagr syndrome. N. Engl. J. Med..

[43] Gray J., Yeo G.S., Cox J.J., Morton J., Adlam A.L., Keogh J.M., Yanovski J.A., El Gharbawy A., Han J.C., Tung Y.C. (2006). Hyperphagia, severe obesity, impaired cognitive function, and hyperactivity associated with functional loss of one copy of the brain-derived neurotrophic factor (BDNF) gene. Diabetes.

[44] Lommatzsch M., Zingler D., Schuhbaeck K., Schloetcke K., Zingler C., Schuff-Werner P., Virchow J.C. (2005). The impact of age, weight and gender on BDNF levels in human platelets and plasma. Neurobiol. Aging.

[45] Radka S.F., Holst P.A., Fritsche M., Altar C.A. (1996). Presence of brain-derived neurotrophic factor in brain and human and rat but not mouse serum detected by a sensitive and specific immunoassay. Brain Res..

[46] Amadio P., Sandrini L., Ieraci A., Tremoli E., Barbieri S.S. (2017). Effect of clotting duration and temperature on BDNF measurement in human serum. Int. J. Mol. Sci..

[47] Fujimura H., Altar C.A., Chen R., Nakamura T., Nakahashi T., Kambayashi J., Sun B., Tandon N.N. (2002). Brain-derived neurotrophic factor is stored in human platelets and released by agonist stimulation. Thromb. Haemost..

[48] Amadio P., Baldassarre D., Sandrini L., Weksler B.B., Tremoli E., Barbieri S.S. (2017). Effect of cigarette smoke on monocyte procoagulant activity: Focus on platelet-derived brain-derived neurotrophic factor (BDNF). Platelets.

[49] Serra-Millàs M. (2016). Are the changes in the peripheral brain-derived neurotrophic factor levels due to platelet activation?. World J. Psychiatry.

[50] Kishino A., Katayama N., Ishige Y., Yamamoto Y., Ogo H., Tatsuno T., Mine T., Noguchi H., Nakayama C. (2001). Analysis of effects and pharmacokinetics of subcutaneously administered BDNF. Neuroreport.

[51] Polyakova M., Schlögl H., Sacher J., Schmidt-Kassow M., Kaiser J., Stumvoll M., Kratzsch J., Schroeter M.L. (2017). Stability of BDNF in human samples stored up to 6 months and correlations of serum and edta-plasma concentrations. Int. J. Mol. Sci..

[52] Trajkovska V., Marcussen A.B., Vinberg M., Hartvig P., Aznar S., Knudsen G.M. (2007). Measurements of brain-derived neurotrophic factor: Methodological aspects and demographical data. Brain Res. Bull..

[53] Tsuchimine S., Sugawara N., Ishioka M., Yasui-Furukori N. (2014). Preanalysis storage conditions influence the measurement of brain-derived neurotrophic factor levels in peripheral blood. Neuropsychobiology.

[54] Eyileten C., Kaplon-Cieslicka A., Mirowska-Guzel D., Malek L., Postula M. (2017). Antidiabetic effect of brain-derived neurotrophic factor and its association with inflammation in type 2 diabetes mellitus. J. Diabetes Res..

[55] Krabbe K.S., Nielsen A.R., Krogh-Madsen R., Plomgaard P., Rasmussen P., Erikstrup C., Fischer C.P., Lindegaard B., Petersen A.M., Taudorf S. (2007). Brain-derived neurotrophic factor (BDNF) and type 2 diabetes. Diabetologia.

[56] Noble E.E., Billington C.J., Kotz C.M., Wang C. (2011). The lighter side of BDNF. Am. J. Physiol. Regul. Integr. Comp. Physiol..

[57] Iughetti L., Casarosa E., Predieri B., Patianna V., Luisi S. (2011). Plasma brain-derived neurotrophic factor concentrations in children and adolescents. Neuropeptides.

[58] Hashimoto K. (2016). Ethnic differences in the serum levels of probdnf, a precursor of brain-derived neurotrophic factor (BDNF), in mood disorders. Eur. Arch. Psychiatry Clin. Neurosci..

[59] Akbarian S.A., Salehi-Abargouei A., Pourmasoumi M., Kelishadi R., Nikpour P., Heidari-Beni M. (2017). Association of brain-derived neurotrophic factor gene polymorphisms with body mass index: A systematic review and meta-analysis. Adv. Med. Sci..

[60] Yeebo M.F. (2015). Ethnic differences in BDNF val66met polymorphism. Br. J. Psychiatry.

[61] Zuccato C., Marullo M., Vitali B., Tarditi A., Mariotti C., Valenza M., Lahiri N., Wild E.J., Sassone J., Ciammola A. (2011). Brain-derived neurotrophic factor in patients with huntington’s disease. PLoS ONE.

[62] Polacchini A., Metelli G., Francavilla R., Baj G., Florean M., Mascaretti L.G., Tongiorgi E. (2015). A method for reproducible measurements of serum BDNF: Comparison of the performance of six commercial assays. Sci. Rep..

[63] Marosi K., Mattson M.P. (2014). BDNF mediates adaptive brain and body responses to energetic challenges. Trends Endocrinol. Metab..

[64] Di Minno A., Turnu L., Porro B., Squellerio I., Cavalca V., Tremoli E., Di Minno M.N. (2016). 8-hydroxy-2-deoxyguanosine levels and cardiovascular disease: A systematic review and meta-analysis of the literature. Antioxid. Redox Signal..

[65] Moher D., Liberati A., Tetzlaff J., Altman D.G. (2009). Preferred reporting items for systematic reviews and meta-analyses: The prisma statement. PLoS Med..

[66] Faraone S.V. (2008). Interpreting estimates of treatment effects: Implications for managed care. P T.

[67] Higgins J.P., Thompson S.G., Deeks J.J., Altman D.G. (2003). Measuring inconsistency in meta-analyses. BMJ.

[68] Sterne J.A., Egger M., Smith G.D. (2001). Systematic reviews in health care: Investigating and dealing with publication and other biases in meta-analysis. BMJ.

[69] Duval S., Tweedie R. (2000). Trim and fill: A simple funnel-plot-based method of testing and adjusting for publication bias in meta-analysis. Biometrics.

[70] Wells G., Shea B., O’Connell D., Peterson J., Welch V., Losos M., Tugwell P. (2000). The Newcastle–Ottawa Scale (Nos) for Assessing the Quality of Non-Randomized Studies in Meta-Analysis.

