# Supplementary material for: Association between Obesity and Circulating Brain-Derived Neurotrophic Factor (BDNF) Levels: Systematic Review of Literature and Meta-Analysis
Source: Int J Mol Sci. 2018 Aug 3;19(8):2281. doi: 10.3390/ijms19082281 (PMC6121551; doi:10.3390/ijms19082281)
Supplement: Supplementary file 1 [file ijms-19-02281-s001.zip › Table S1.pdf]

Table S1: Demographic and clinical data of obese patients and healthy controls of the included studies.

Author, Country	TC (mg/dl)	TC (mg/dl) CTRLs	TC (mg/dl) Weighted average	HDL (mg/dl)	HDL (mg/dl) CTRLs	HDL (mg/dl) Weighted average	LDL (mg/dl)	LDL (mg/dl) CTRLs	LDL (mg/dl) Weighted average	TG (mg/dl)	TG (mg/dl) CTRLs	TG (mg/dl) Weighted average	Glycaemia (mg/dl)	Glycaemia (mg/dl) CTRLs	Glycaemia (mg/dl) Weighted average
Lee I-Te 2016, Taiwan	201	186	195	39	54	45	ND	ND	ND	283	97	207	101	90	96
Slusher AL 2015, United States	ND	ND	ND	ND	ND	ND	ND	ND	ND	ND	ND	ND	98	89	94
Hinderberger P 2016, Germany	ND	ND	ND	ND	ND	ND	ND	ND	ND	ND	ND	ND	ND	ND	ND
Gajewska E 2014, Poland	205,7	246,6	223	45,2	45,4	45,3	127,8	158,4	140,7	162,9	221,6	188	107,5	93,2	101
	233,8	232,6	233,3	39,2	49,3	43,1	158	151	155,3	183,2	160	174	104,8	99	103
	216,1	172,2	199,9	34,4	41,2	36,9	139,2	103,2	125,9	139,2	139	139	101	97,8	100
	220,8	208	213,1	35,5	43,8	40,5	144,7	132,8	137,6	164,8	153,7	158	96,9	92,4	94
Han JC 2010, United States	ND	ND	ND	ND	ND	ND	ND	ND	ND	ND	ND	ND	ND	ND	ND
Corripio R 2012, Spain	155,5	157,8	156	51,3	65,5	57	93,5	82,6	89	ND	ND	ND	83,7	83,3	84
Lee I-Te 2012, Taiwan	201	186	195	39	54	45	ND	ND	ND	213	97	165	97	90	94
Lee SS 2014, Korea	ND	ND	ND	ND	ND	ND	ND	ND	ND	ND	ND	ND	ND	ND	ND
Levinger I 2007, Australia	ND	ND	ND	54	62	57	ND	ND	ND	124	62	98	86	101	92
Chun-Jung Huang 2013, United States	ND	ND	ND	ND	ND	ND	ND	ND	ND	ND	ND	ND	ND	ND	ND

TC: Total cholesterol, HDL: High density lipoproteins, LDL: Low density lipoproteins, TG: Triglycerides, ND: Not Declared
