# Supplementary figures and images for: Association between Obesity and Circulating Brain-Derived Neurotrophic Factor (BDNF) Levels: Systematic Review of Literature and Meta-Analysis
Source: Int J Mol Sci. 2018 Aug 3;19(8):2281. doi: 10.3390/ijms19082281 (PMC6121551; doi:10.3390/ijms19082281)

S2 Fig. Scatterplot of the relationship between male gender and age and mean difference.

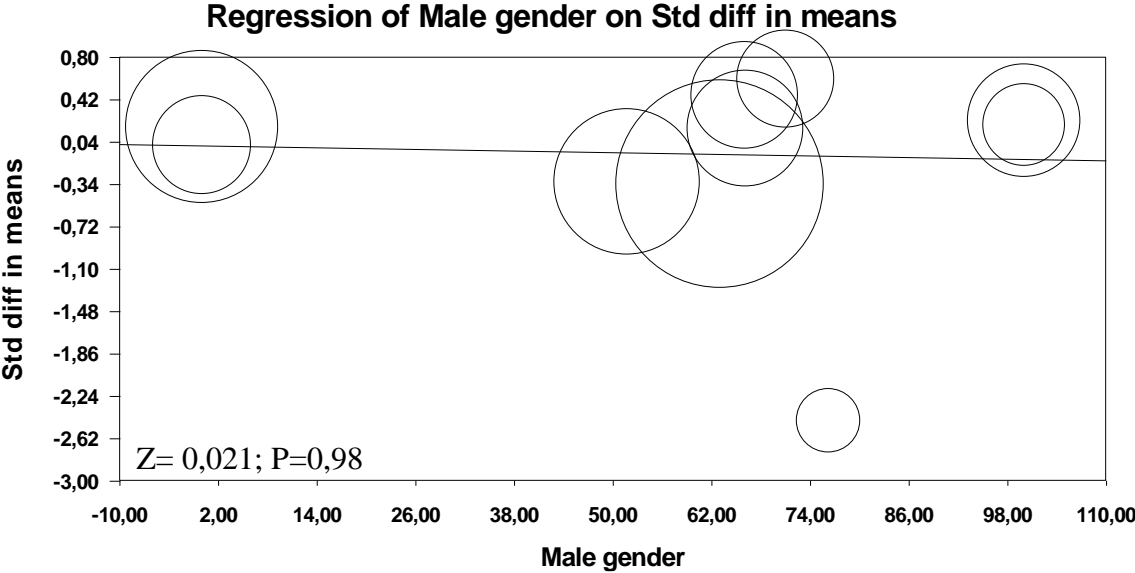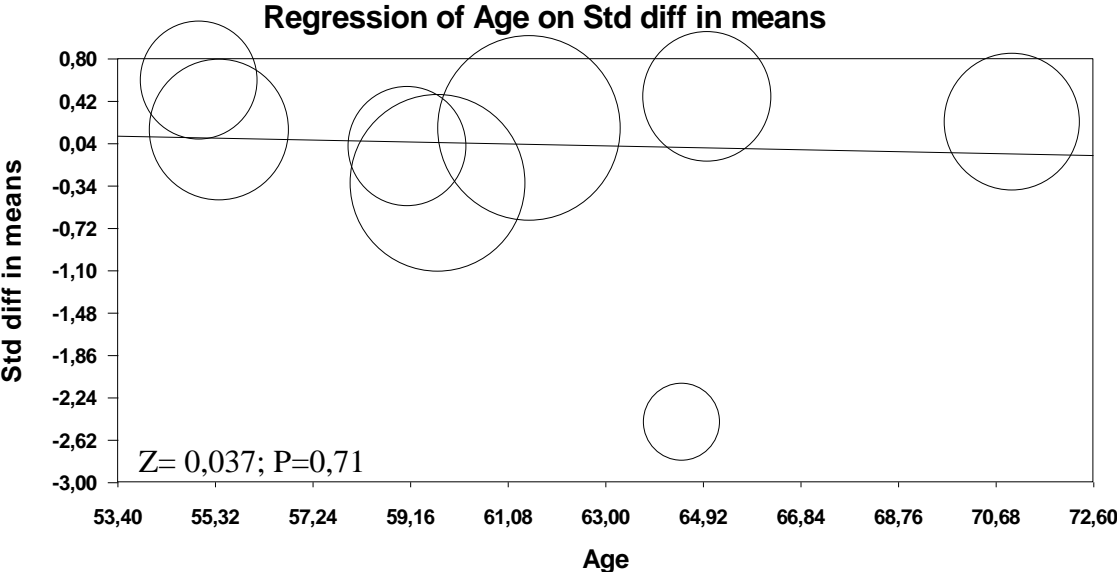

Supplement: Supplementary file 1 [file ijms-19-02281-s001.zip › Figure S2.pdf]
